# Impact of Gentamicin Concentration and Exposure Time on Intracellular *Yersinia pestis*

**DOI:** 10.3389/fcimb.2017.00505

**Published:** 2017-12-11

**Authors:** Tiva T. VanCleave, Amanda R. Pulsifer, Michael G. Connor, Jonathan M. Warawa, Matthew B. Lawrenz

**Affiliations:** Department of Microbiology and Immunology and Center for Predictive Medicine for Biodefense and Emerging Infectious Diseases, University of Louisville School of Medicine, Louisville, KY, United States

**Keywords:** *Yersinia pestis*, antibiotic protection assay, gentamicin, intracellular survival, macrophages

## Abstract

The study of intracellular bacterial pathogens in cell culture hinges on inhibiting extracellular growth of the bacteria in cell culture media. Aminoglycosides, like gentamicin, were originally thought to poorly penetrate eukaryotic cells, and thus, while inhibiting extracellular bacteria, these antibiotics had limited effect on inhibiting the growth of intracellular bacteria. This property led to the development of the antibiotic protection assay to study intracellular pathogens *in vitro*. More recent studies have demonstrated that aminoglycosides slowly penetrate eukaryotic cells and can even reach intracellular concentrations that inhibit intracellular bacteria. Therefore, important considerations, such as antibiotic concentration, incubation time, and cell type need to be made when designing the antibiotic protection assay to avoid potential false positive/negative observations. *Yersinia pestis*, which causes the human disease known as the plague, is a facultative intracellular pathogen that can infect and replicate in macrophages. *Y. pestis* is sensitive to gentamicin and this antibiotic is often employed in the antibiotic protection assay to study the *Y. pestis* intracellular life cycle. However, a large variety of gentamicin concentrations and incubation periods have been reported in the *Y. pestis* literature without a clear characterization of the potential influences that variations in the gentamicin protection assay could have on intracellular growth of this pathogen. This raised concerns that variations in the gentamicin protection assay could influence phenotypes and reproducibility of data. To provide a better understanding of the potential consequences that variations in the gentamicin protection assay could have on *Y. pestis*, we systematically examined the impact of multiple variables of the gentamicin protection assay on *Y. pestis* intracellular survival in macrophages. We found that prolonged incubation periods with low concentrations of gentamicin, or short incubation periods with higher concentrations of the antibiotic, have a dramatic impact on intracellular growth. Furthermore, the degree of sensitivity of intracellular *Y. pestis* to gentamicin was also cell type dependent. These data highlight the importance to empirically establish cell type specific gentamicin protection assays to avoid potential artificial data in *Y. pestis* intracellular studies.

## Introduction

Many bacterial pathogens have evolved mechanisms to infect and survive within host cells. Therefore, in order to study and understand the pathogenesis of intracellular pathogens, cell culture-based *in vitro* assays have been developed. For obligate intracellular pathogens, like *Chlamydia* species, which cannot grow outside of a host cell, these assays are relatively straight forward. Bacteria are added directly to cells, allowed to adhere and invade for a certain period of time, and the remaining extracellular bacteria are removed by washing the cells (Kokes and Valdivia, 2015; Zuck et al., 2017). However, for facultative intracellular bacteria that can also replicate in tissue culture medium, additional steps are needed to inhibit the growth of extracellular bacteria not removed by washing. The most common approach to limit extracellular growth is to include an antibiotic to the medium after bacteria have had time to invade cells. Importantly, the antibiotic chosen should have a limited ability to cross the plasma membrane of the cell. Thus, intracellular bacteria are protected from the antibiotic while extracellular bacteria are inhibited by the antibiotic (commonly referred to as an antibiotic protection assay) (Utili et al., 1991; Tabrizi and Robins-Browne, 1993). Application of the antibiotic protection assay have allowed the study of intracellular virulence mechanisms across multiple facultative intracellular bacteria (Kaneko et al., 2016).

While antibiotic protection assay are commonly used to study intracellular pathogens, strong evidence indicates that antibiotics originally thought to be completely excluded by the host cell can slowly enter and accumulate within cells. For example, aminoglycosides such as streptomycin and gentamicin are often employed in the antibiotic protection assay because they are not lipid soluble and original studies indicating poor cell permeability (Bonventre et al., 1967; Prokesch and Hand, 1982; Hand and King-Thompson, 1986). However, subsequent studies have demonstrated that despite poor membrane permeability, aminoglycosides can still accumulate within cells to levels that inhibit bacterial growth, especially for organisms that remain in vacuolar compartments. Such phenomenon has been reported for antibiotic protection assays involving *Listeria monocytogenes, Salmonella enterica, Staphylococcus aureus*, and *E. coli* (Drevets et al., 1994; Hamrick et al., 2003; Menashe et al., 2008; Flannagan et al., 2016). The increased sensitivity of organisms within phagosomal compartments to aminoglycosides, as opposed to those that escape into the cytoplasm, has been attributed to the believed primary route of antibiotic uptake—pinocytosis of the extracellular milieu (Drevets et al., 1994). Pinocytosed vesicles containing the antibiotic in turn readily fuse with phagosomes/endosomes, delivering the aminoglycoside to the bacterial containing compartment. However, it should be noted that aminoglycosides have also been shown to accumulate in the cytosol depending on cell type used and length of incubation time with the antibiotic, and thus, addition of aminoglycosides may also artificially influence intracellular growth of cytoplasmic pathogens (Drevets et al., 1994; Myrdal et al., 2005). Therefore, these data should be taken into consideration when designing antibiotic protection assays to study intracellular growth.

*Yersinia pestis*, the causative agent of plague, is a facultative intracellular pathogen that is able to invade and replicate in host cells (Burrows and Bacon, 1956; Cavanaugh and Randall, 1959; Straley and Harmon, 1984a; Pujol and Bliska, 2003, 2005; Grabenstein et al., 2006; Spinner et al., 2014). Of particular interest has been *Y. pestis* intracellular survival in macrophages (Pujol and Bliska, 2005; Bliska and Casadevall, 2009). However, *Y. pestis* has also been shown to survive in epithelial cells (Cowan et al., 2000) and more recently, within neutrophils (O'Loughlin et al., 2010; Spinner et al., 2013, 2014; Shannon et al., 2015). As *Y. pestis* does not express many extracellular virulence factors in the flea vector, it is believed that intracellular survival is important during the early stages of infection after transmission from the flea, and provides a protective niche for the bacterium to initiate expression of the factors needed to exist extracellularly (Cavanaugh and Randall, 1959; Hinnebusch, 2005; Oyston and Isherwood, 2005; Pujol and Bliska, 2005; Grabenstein et al., 2006). Once *Y. pestis* invades a cell, it is able to generate a replicative niche within a phagosomal compartment (called the *Yersinia* containing vacuole or YCV) by subverting the normal maturation of the phagosome (Straley and Harmon, 1984b; Grabenstein et al., 2004; Pujol et al., 2009; Connor et al., 2015). The YCV appears to be maintained throughout the intracellular infection until the host cell eventually lyses, releasing the bacteria into the extracellular environment (Pujol et al., 2009).

As *Y. pestis* is able to grow in most cell culture media, in order to study intracellular interactions with host cells *in vitro*, the antibiotic protection assay is needed to inhibit extracellular growth. In most cases gentamicin is the primary antibiotic employed for *Y. pestis* intracellular studies because the bacterium is sensitive to the antibiotic (MIC ~2 μg/ml) (Smith et al., 1995). Surprisingly, in light of the potential influence gentamicin has on the intracellular growth of other bacteria, a large variety of gentamicin concentrations (ranging from 0.016 to 256 μg/ml) and incubation times (from 15 min to 2 h) have been reported in the *Y. pestis* literature (Pujol and Bliska, 2003; Benedek et al., 2004; Leigh et al., 2005; Ponnusamy et al., 2011; Sha et al., 2013; Spinner et al., 2014; Tiner et al., 2015; van Lier et al., 2015). Not surprisingly, variation in *Y. pestis* intracellular survival has also been observed. Importantly, characterization of potential *Y. pestis* pathogenesis factors involved in intracellular infection, or attempts to repeat published data, could be influenced simply by the concentration of gentamicin used in the antibiotic protection assay. Therefore, we felt it was imperative to understand the potential influence of gentamicin concentrations on *Y. pestis* intracellular growth in order to eliminate any potential for unintended influence of the gentamicin protection assay on the interpretation of intracellular studies with *Y. pestis*.

## Materials and methods

### Cell culture and bacterial strains

Primary peritoneal macrophages were harvested from 8 to 12 week old C57/Bl6 mice (University of Louisville IACUC Protocols 16651 and 16723) injected with 3 mls of sterile Brewer's thioglycolate medium. Four days after injection, mice were humanely euthanized and peritoneal macrophages were recovered in 20 mls of Hank's Buffered Salt Solution (HBSS). Cells were isolated by centrifugation and resuspended in 10 mls of DMEM (DMEM, 100 mM glucose, sodium pyruvate; Hyclone) + 10% FBS (Biowest) as previously described (Ray and Dittel, 2010). Cells were quantified, transferred to microtiter plates or dishes, and allowed to adhere for 3 h. Non-adherent cells were then removed from adherent macrophages by washing with HBSS three times. RAW264.7 macrophages were originally obtained from ATCC and cultured in DMEM + 10% FBS. RAW264.7 cells were propagated for only up to 15 passages for these studies. *Y. pestis* strains used in these studies are listed in Table 1. Bacteria were propagated in Difco Brain Heart Infusion (BHI) (BD, Co.). We have previously shown that the bioluminescence generated by the Lux_P*tolC*_ bioreporter directly correlates with bacterial numbers and can be used to kinetically monitor *Y. pestis* intracellular survival in gentamicin protection assays (Sun et al., 2012; Connor et al., 2015).

**Table 1:** *Yersinia pestis* strains used in this study.

**Strain**	**Relevant Characteristics**	**Bioreporter**	**Source**
YPA165	CO92 pgm^(−)^ pCD1^(+)^	pGEN222::mCherry	This study
YPA081	CO92 pgm^(+)^ pCD1^(−)^	pGEN222::EGFP	Connor et al., 2015
YPA050	CO92 pgm^(+)^ pCD1^(−)^	Lux_P*tolC*_	Sun et al., 2012
YP043	CO92 pgm^(+)^ pCD1^(+)^	Lux_P*tolC*_	Sun et al., 2012

### Live cell microscopy

5 × 10^5^ peritoneal macrophages in FluoroBrite DMEM (ThermoFisher), 4 mM glutamine + 10% FBS were added to a 35 mm glass bottom FluoroDish (World Precision Instruments). Macrophages were allowed to adhere to the dish for 3 h prior to infection. *Y. pestis* was grown overnight in BHI broth at 26°C and then diluted 1:25 in fresh BHI broth and grown for an additional 3 h until the culture reached an absorbance at 600 nm of ~1.0. The bacteria were diluted into FluoroBrite DMEM, 4 mM glutamine + 10% FBS and added to macrophages at a multiplicity of infection (MOI) of 3 bacteria per macrophage. To facilitate cell-bacteria interactions, FluoroDishes were centrifuged at 200 × g for 5 min and returned to the CO_2_ incubator. Fifteen minutes later, the medium was removed, the cells were washed three times with sterile 1X PBS to remove extracellular bacteria, and fresh FluoroBrite DMEM, 4 mM glutamine + 10% FBS was added. Live imaging was performed using a Nikon A1 confocal microscope (Nikon Instruments, Inc.) with a live cell chamber equilibrated to 5% CO_2_ and 37°C prior to imaging. Either a 488 nm or 561 nm laser with filter sets 525 ± 50 nm or 595 ± 50 nm were used to visualize EGFP or mCherry, respectively. Images were taken using a 40x Plan Fluro Oil objective every 20 min beginning 1 h post-infection and continuing for 24 h. The Nikon Perfect Focus System and field tiling were used to collect six fields (287 × 287 μM per field) at each time point. Bacterial numbers were estimated by calculating the area of the fluorescent signal for each field using FIJI (Schindelin et al., 2012).

### Monitoring bacteria using bioluminescence

1.5 × 10^5^ macrophages were aliquoted into the wells of a white 96 well microtiter plate (Greiner Bio One) and allowed to adhere for 3 or 15 h (peritoneal or RAW264.7 cells, respectively). *Y. pestis* was grown overnight in BHI broth at 26°C and then diluted 1:25 in fresh BHI broth and grown for an additional 3 h until the culture reached an absorbance at 600 nm of ~1.0. The bacteria were diluted into 37°C DMEM + 10% FBS and added to macrophages at a MOI of 10 bacteria per macrophage. To facilitate cell-bacteria interactions, microtiter plates were centrifuged at 200 × g for 5 min and returned to the CO_2_ incubator for an additional 15 min, at which point gentamicin was added directly to the wells without washing to achieve the final desired concentrations. Concentrations of gentamicin used were not cytotoxic to macrophages. Bacterial numbers, as a function of bioluminescence produced by the Lux_P*tolC*_ bioreporter (Sun et al., 2012; Connor et al., 2015), were monitored kinetically to limit manipulation of the macrophages using a Synergy HT plate reader (0.5 s read, sensitivity of 135) (BioTek) or IVIS Spectrum camera system (5 sec with medium binning through a 500 nm emission filter) (Caliper).

### Determination of gentamicin minimal inhibitory concentration

*Y. pestis* was grown overnight in BHI broth at 26°C and then diluted 1:25 in fresh BHI broth and grown for an additional 3 h until the culture reached an absorbance at 600 nm of ~1.0. The bacteria were diluted into 37°C DMEM + 10% FBS and aliquoted at 1.5 × 10^6^ CFU per well in a 96 well plate. Gentamicin was diluted in 37°C DMEM + 10% FBS and added to the bacteria to achieve the final desired concentration (0–128 μg/ml). One h after treatment, bacterial numbers were determined by conventional enumeration using serial dilution and plating on BHI agar (Sun et al., 2012).

### Enumerating intracellular bacteria by conventional enumeration

For short incubations with the antibiotic, bacterial numbers were directly enumerated by serial dilution to allow for separate calculations of extracellular and intracellular bacterial numbers after gentamicin treatment. Macrophages were infected with *Y. pestis* as described above and treated with gentamicin for 1 h. After treatment with gentamicin, the culture medium was collected into a separate tube, the cells were washed three times with sterile 1X PBS, which was also collected and combined with the collected medium, and the combined medium + 1x PBS washes was serial diluted and plated on BHI agar plates to enumerate the extracellular bacteria. For the intracellular bacteria, washed macrophages were lysed with 0.1% Triton X-100 and serial dilutions were plated on BHI agar plates for enumeration (Sun et al., 2012).

### Statistical analysis

All studies were repeated three times to ensure reproducibility. When needed, mean values from individual treatment groups were compared to the 0 μg/ml gentamicin group using the ANOVA with the Dunnett's *post-test* or across all samples with the Tukey *post-test*. A *p*-value <0.5 was consider to be statistically significant.

## Results

### Macrophage—*Y. pestis* interactions in the absence of gentamicin

**Figure 1:**
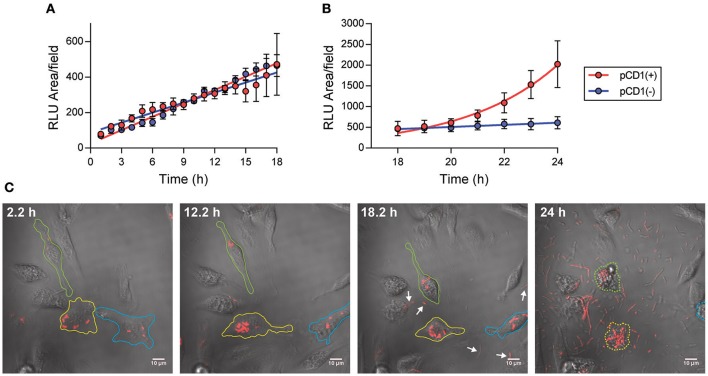
Live imaging of *Y. pestis* in the absence of gentamicin. **(A,B)** Primary peritoneal macrophages were infected with *Y. pestis* YPA165 expressing mCherry [red symbols; pCD(+)] or *Y. pestis* YPA081expressing EGFP [blue symbols; pCD(–)] at a MOI = 3. One h after infection, cells were washed to remove extracellular bacteria and macrophages were imaged by live confocal microscopy. Bacterial numbers as a function of total fluorescent (RLU) area per field were determined every 1 h for 24 h. Each point represents the mean ± *S.D*. calculated from four imaged fields at each time point (an average of 16 infected cells per field). **(C)** Representative images of *Y. pestis* YPA165 infected macrophages at indicated time points during the infection. Individual macrophages tracked through the entire course of the experiment are outlined in different colors. Lysed macrophages at 24 h are highlighted by dotted lines. Presence of extracellular bacteria at 18.2 h are highlighted by arrows.

To establish the fate of *Y. pestis* during infection of macrophages in the absence of gentamicin treatment, primary peritoneal macrophages were infected with a *Y. pestis* strain expressing mCherry fluorescent protein (YPA165). Twenty minutes post-infection, macrophages were washed to remove extracellular bacteria and imaged every 20 min by laser confocal microscopy for 24 h (Supplemental Movie 1). Changes in bacterial number, as a function of the total area of mCherry signal, were calculated at each time point (Figure 1). Over the first ~12 h, most bacteria appeared to be intracellular and we observed a steady increase in the area of mCherry over time, indicating intracellular survival and growth (Figure 1A). However, a small number of infected macrophages began to lyse ~12 h post-infection and release *Y. pestis* into the culture medium, though cell death was not synchronous and most infected cells were still intact through the first 18 h (Figure 1C). We continued to observe increases in the area of mCherry through 24 h of observation (Figure 1B), and while bacteria appeared to continue to grow intracellularly, extracellular bacteria released from dead macrophages were also replicating in the medium and represented a large portion of the mCherry signal at later time points (Figures 1B,C, (Supplemental Movie 1). By 24 h post-infection, almost all of the infected macrophages had lysed and the majority of the mCherry signal was from extracellular bacteria (Figure 1C, (Supplemental Movie 1).

To determine if the *Y. pestis* Ysc type 3 secretion system (T3SS) influenced the intracellular fate of the bacterium, macrophages were infected with a *Y. pestis* strain lacking the pCD1 virulence plasmid encoding the Ysc T3SS and expressing EGFP (YPA081) and imaged by laser confocal microscopy as described above (Supplemental Movie 2). As observed for the T3SS positive strain, total area of EGFP increased over the entire course of the experiment (Figures 1A,B). However, unlike the *Y. pestis* pCD1^(+)^ strain, we did not observe macrophage lysis, bacterial release, or extracellular proliferation over the 24 h time frame of the experiment, indicating that the increased area of EGFP was a function of only intracellular growth. Together these data: (1) confirm data from previous *in vitro* assays that *Y. pestis* survives intracellularly in primary macrophages (Straley and Harmon, 1984a,b); (2) confirm that *Y. pestis* lacking the T3SS can survive in macrophages (Straley and Harmon, 1984a,b; Pujol and Bliska, 2003); (3) demonstrate that macrophages infected with *Y. pestis* with a functional T3SS lyse more rapidly than macrophages infected with *Y. pestis* lacking the T3SS; (4) macrophage cell death releases intracellular bacteria into the environment; and (5) bacteria released from macrophages are viable and can replicate in the cell culture medium. While intracellular vs. extracellular bacteria can be differentiated during live imaging, *in vitro* assays that do not use microscopy are not able to differentiate between the two, thus requiring the use of an antibiotic protection assay. However, as a wide variety of concentrations of gentamicin have been reported in the *Yersinia* literature (Pujol and Bliska, 2003; Benedek et al., 2004; Leigh et al., 2005; Ponnusamy et al., 2011; Sha et al., 2013; Spinner et al., 2014; Tiner et al., 2015; van Lier et al., 2015), we next wanted to determine if extended incubation with gentamicin could influence intracellular proliferation of *Y. pestis*.

### Extended incubation with gentamicin impacts intracellular survival of *Y. pestis*

**Figure 2:**
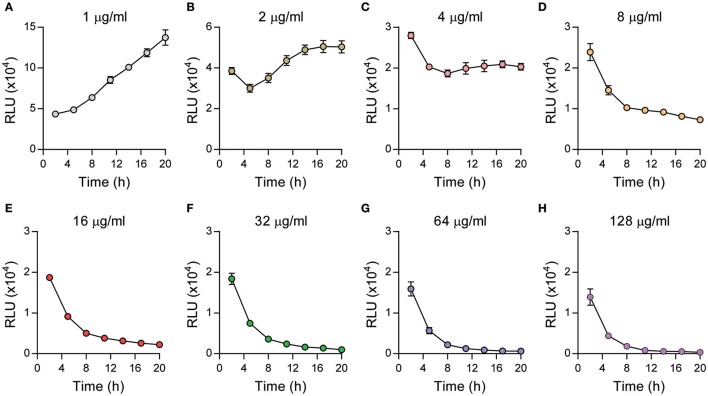
Extended incubations with gentamicin inhibit *Y. pestis* intracellular survival. Primary peritoneal macrophages were infected with *Y. pestis* YPA050 at an MOI of 10 (*n* = 6). 20 min post-infection, macrophages were washed and the medium was replaced with medium containing gentamicin at indicated concentrations **(A–H)**. Bacterial numbers as a function of bioluminescence were determined every 3 h for 20 h using a plate reader and displayed as the mean ± *S.D*. (in some cases the *S.D*. is smaller than the symbol size). Data is shown from one representative experiment of three independent experiments.

The simplest antibiotic protection assay would be to add gentamicin to the infected cells and maintain that concentration in the medium throughout the entire course of the study. However, extended incubations have been shown to influence intracellular proliferation of other bacteria (Utili et al., 1991; Drevets et al., 1994; Menashe et al., 2008). To determine if extended incubations with gentamicin impacted survival of intracellular *Y. pestis*, peritoneal macrophages were infected with a *Y. pestis* strain containing the Lux_P*tolC*_ bioreporter (YPA050), which we have previously shown can be used to accurately monitor intracellular *Y. pestis* as a function of bioluminescence (Sun et al., 2012; Connor et al., 2015). Twenty minutes post-infection, serial dilutions of gentamicin were added to each well to achieve final concentrations of 128 to 1 μg/ml and beginning 2 h after infection bacterial numbers, as a function of bioluminescence, were determined every 3 h for 20 h using a plate reader. We observed that at the lowest concentrations tested, 1 and 2 μg/ml, bacterial bioluminescence increased over time, although at 2 μg/ml, bioluminescence was approximately 2–3-fold lower than at 1 μg/ml (Figures 2A,B). At concentrations greater than 2 μg/ml, bioluminescence no longer increased, indicating that bacteria were no longer able to proliferate. At 4 μg/ml, we observed an initial decrease in bioluminescence between 2 and 5 h, at which time bioluminescence remained constant over the remainder of the assay (Figure 2C). Gentamicin concentrations greater than 4 μg/ml resulted in a steady decrease in bacterial bioluminescence over the entire course of the assay (Figures 2D–H). Compared to our live imaging experiments without gentamicin, these data demonstrate that extended incubation with even low concentrations of gentamicin can have a dramatic influence on *Y. pestis* intracellular survival, and can lead to intracellular killing of the bacterium.

### Sensitivity to gentamicin is cell type dependent

**Figure 3:**
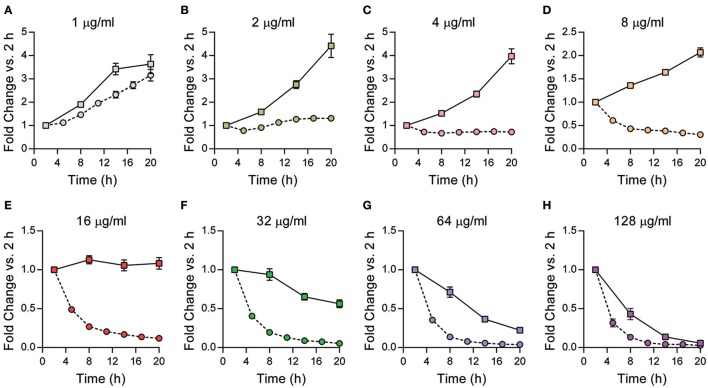
Cell type can influence intracellular sensitivity of *Y. pestis* to gentamicin. Primary peritoneal macrophages (circles, dotted lines) or RAW264.7 macrophages (squares, solid lines) were infected with *Y. pestis* YPA050 at an MOI of 10 (*n* = 6). 20 min post-infection, macrophages were washed and the medium was replaced with medium containing gentamicin at indicated concentrations **(A–H)**. Bacterial numbers as a function of bioluminescence were determined every 3–6 h for 20 h using a plate reader. Bioluminescent data from each time point was normalized to fold change compared to the 2 h time point by dividing each time point by the 2 h RLU reading and is displayed as mean fold change ± *S.D*. Data is shown from one representative experiment of three independent experiments.

As pinocytosis is suggested to be the main mechanism for gentamicin uptake by macrophages, sensitivity of intracellular bacteria to gentamicin could vary depending on cell type used. RAW264.7 cells are an immortal cell line originally derived from mouse macrophages and are commonly used as a macrophage model (Raschke et al., 1978). To determine if the sensitivity of intracellular *Y. pestis* to gentamicin could vary depending on cell type, RAW264.7 cells were infected with *Y. pestis* YPA050. Twenty minutes later, serial dilutions of gentamicin were added to each well to achieve final concentrations of 128 to 1 μg/ml and cells were incubated for 20 h. Bacterial numbers were monitored as a function of bioluminescence, normalized to *T* = 2 h, and compared to similarly treated primary peritoneal macrophages (Figure 3). Similar to peritoneal cells, bacterial bioluminescence increased in RAW264.7 macrophages when treated with 1 μg/ml, indicating intracellular proliferation. However, unlike bacteria in peritoneal macrophages, *Y. pestis* bioluminescence continued to increase in RAW264.7 cells until gentamicin concentrations reached 16 μg/ml. After 16 μg/ml, bioluminescence began to decrease overtime, indicating bacterial killing, and approached levels similar to that seen for peritoneal macrophages at 128 μg/ml. These data highlight that gentamicin inhibition of intracellular *Y. pestis* can vary between cell types.

### Short incubation with high concentrations of gentamicin inhibits intracellular growth

**Figure 4:**
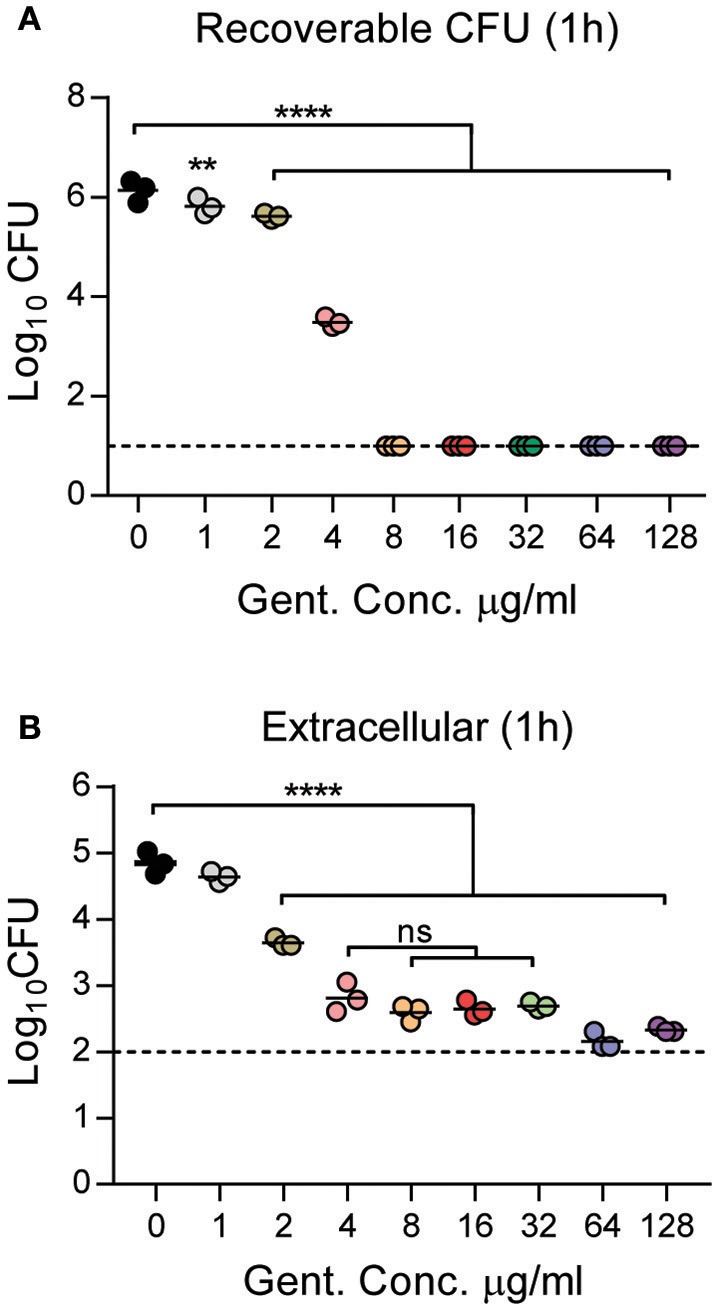
Minimum inhibitory concentration of gentamicin for *Y. pestis*. Logarithmically grown *Y. pestis* YPA050 was diluted to 1.5 × 10^6^ CFU per well in a 96 well plate containing gentamicin at the indicated concentrations. **(A)** One h after inoculation into gentamicin, bacteria were removed, serially diluted in 1 × PBS, and enumerated on agar to determine bacterial viability after exposure to gentamicin (*n* = 3). **(B)** Primary peritoneal macrophages were infected with *Y. pestis* YPA050 at an MOI of 10 (*n* = 3). 20 min post-infection, gentamicin was added at indicated concentrations. One h later, the medium and washes were collected, combined, and serially diluted to enumerate viable bacteria by plating dilutions on BHI agar. Each point represents one sample and the bars represent the mean CFU. The dotted lines indicate the limit of detection. Data is shown from one representative experiment of three independent experiments. **(A,B)** ANOVA with Dunnett's *post-hoc* analysis was used when comparing samples to 0 μg/ml gentamicin. **(B)** ANOVA with Tukey's *post-hoc* analysis was used when comparing between the 4 and 32 μg/ml samples. ^**^p ≤ 0.01; ^****^p ≤ 0.0001; ns = not significant.

Since extended incubations with gentamicin dramatically inhibited the proliferation of intracellular *Y. pestis*, we next sought to identify the minimal concentration of gentamicin needed to inhibit *Y. pestis* growth in tissue culture medium in a 1 h period. Toward this end, *Y. pestis* YPA050 was diluted in DMEM + 10% FBS with increasing concentrations of gentamicin. One h after addition of gentamicin, bacteria were serially diluted and plated on BHI agar to determine remaining viable bacteria (Figure 4A). All doses of gentamicin tested resulted in a significant decrease in the number of recovered bacteria compared to untreated (*p* ≤ 0.0001) and the degree of inhibition was dependent on the concentration of the antibiotic. While bacteria were reduced ~1,000-fold within 1 h with 4 μg/ml gentamicin, no detectable viable bacteria were recovered after incubation with ≥8 μg/ml. Similar inhibition was observed for the Medivalis biovar KIM D19 (Supplemental Figure 1).

Next we sought to determine whether the presence of macrophages impacted the minimal concentration of gentamicin required to inhibit *Y. pestis* growth in cell culture medium. Peritoneal macrophages were infected with *Y. pestis* YPA050 for 20 min and serial dilutions of gentamicin were added to each well to achieve final concentrations of 128 to 1 μg/ml. One h after addition, the gentamicin containing medium was removed and combined with washes and viable extracellular bacteria were determined by serial dilution and conventional enumeration (Figure 4B). Extracellular bacterial numbers were significantly lower in all the samples that received greater than 1 μg/ml of gentamicin as compared to absence of gentamicin (*p* ≤ 0.0001). However, no significant differences were observed in bacterial numbers between concentrations of 4–32 μg/ml. Furthermore, while we observed no significant difference in bacterial numbers between the 64 and 128 μg/ml concentrations, we observed ~2.5-fold lower numbers of extracellular bacteria at 64 and 128 μg/ml samples (average 180 CFU) compared to the 4–32 μg/ml samples (average 458 CFU) that was statistically significant (*p* ≤ 0.05).

**Figure 5:**
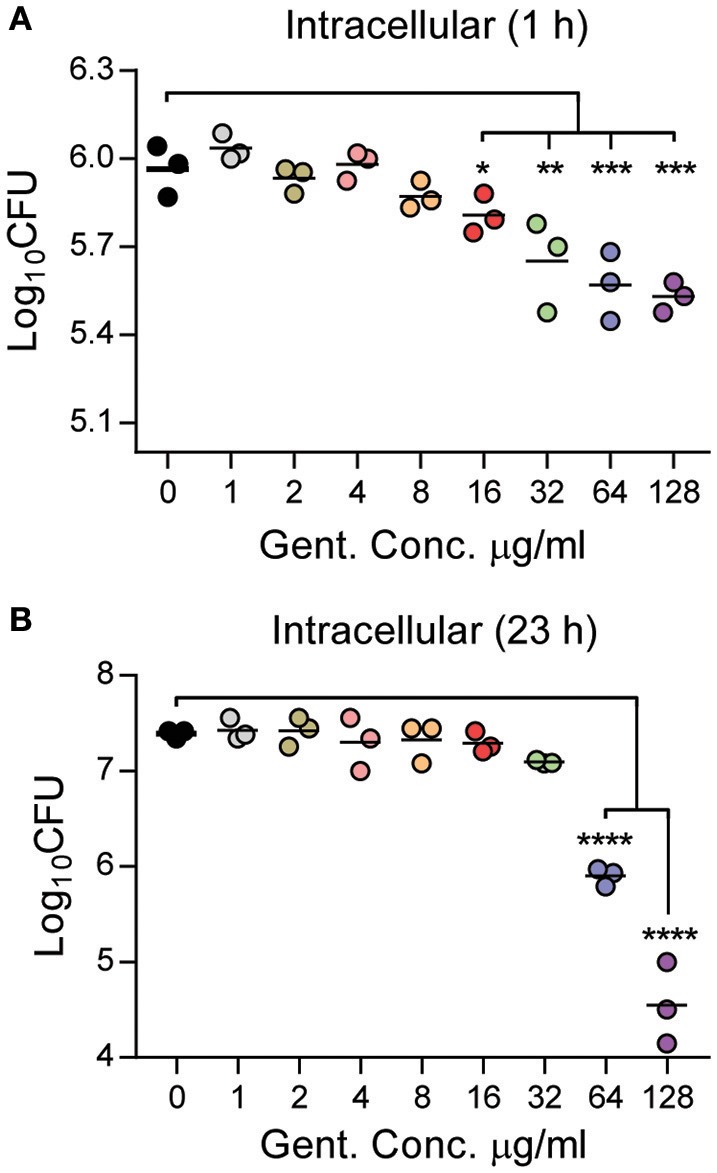
Short incubations with gentamicin impact *Y. pestis* intracellular survival in a dose dependent manner. Primary peritoneal macrophages were infected with *Y. pestis* YPA050 at an MOI of 10 (*n* = 3). Twenty minutes post-infection, the medium was replaced with medium containing gentamicin at indicated concentrations. One h later, gentamicin was removed, and **(A)** cells were washed three times, lysed with 1% Triton, and intracellular bacterial were enumerated by serial dilution and plating on BHI agar plates, or **(B)** fresh medium without gentamicin was added and cells incubated for an additional 23 h. At 23 h post-gentamicin treatment, the medium was removed, cells were washed three times, lysed with 0.1% Triton, and intracellular bacterial were enumerated by serial dilution and plating on BHI agar plates. Each point represents one sample and the bars represent the mean CFU. Data is shown from one representative experiment of three independent experiments. ANOVA with Dunnett's *post-hoc* analysis compared to 0 μg/ml gentamicin: ^*^p ≤ 0.05; ^**^p ≤ 0.01; ^***^p ≤ 0.001; ^****^p ≤ 0.0001.

Finally, we enumerated the number of intracellular bacteria in macrophages after 1 h treatment with gentamicin at both 1 and 24 h post-treatment to determine if gentamicin concentration during short incubations could impact intracellular survival of *Y. pestis* (Figure 5). At 1 h post-treatment, statistically significant differences in the number of intracellular bacteria recovered were not observed until gentamicin concentrations were greater than 8 μg/ml (Figure 5A). In concentrations >8 μg/ml, a dose dependent decrease in viable intracellular bacteria was observed. However, even at the highest doses, intracellular numbers only varied by ~1.3-fold as compared to macrophages receiving lower doses of antibiotic. To determine if long term intracellular survival of *Y. pestis* was affected by short exposures to gentamicin, a separate group of infected cells were washed 1 h after gentamicin treatment to remove residual gentamicin and fresh medium without gentamicin was added. Twenty three hour later, the medium was removed, the cells were washed, lysed, and intracellular bacterial numbers were enumerated (Figure 5B). We observed bacterial proliferation in all samples containing <64 μg/ml gentamicin, and there were no significant differences in intracellular numbers in samples containing 0–32 μg/ml gentamicin. However, bacteria did not proliferate in macrophages treated with 64 μg/ml of the antibiotic, and at the highest dose of 128 μg/ml, intracellular bacterial numbers decreased over time. Together these data indicate that at least 4 μg/ml of gentamicin is required to achieve optimal killing of extracellular *Y. pestis* in 1 h during macrophage infection assays. However, as gentamicin concentrations increase, even short incubations with the antibiotic can impact proliferation of intracellular *Y. pestis*, especially at higher doses of the antibiotic.

### Optimized gentamicin protection assay for *Y. pestis*

**Figure 6:**
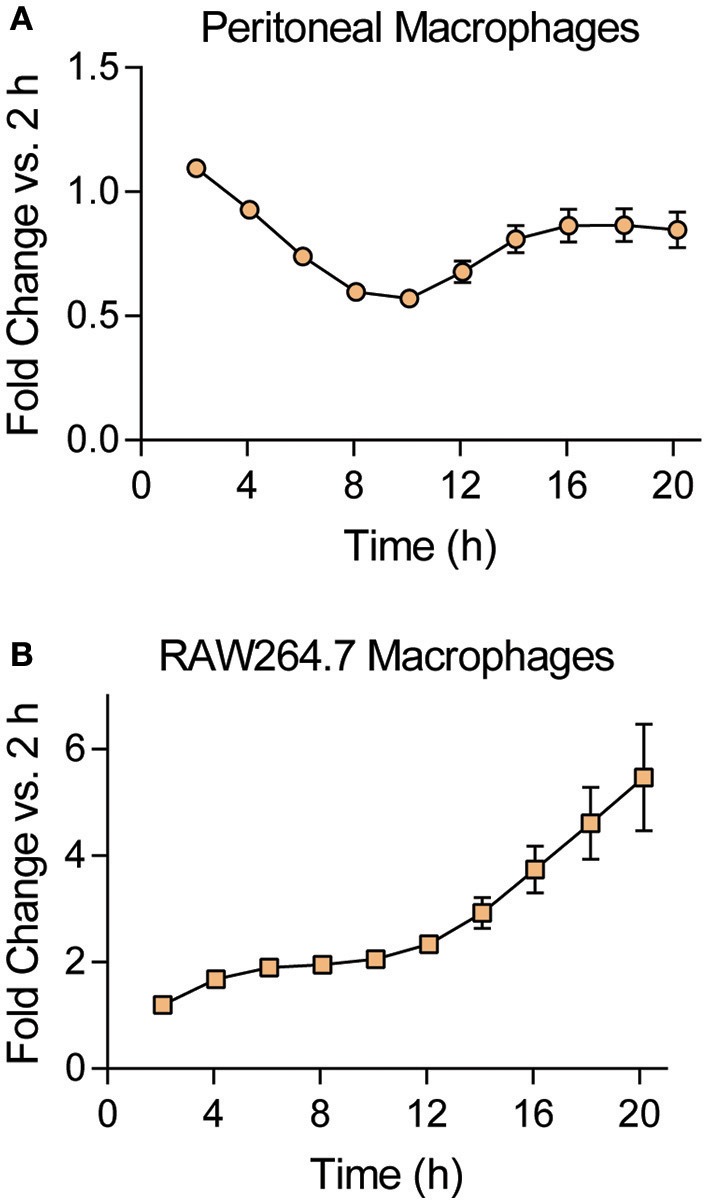
Optimized gentamicin protection assay. **(A)** Primary peritoneal or **(B)** RAW264.7 macrophages were infected with *Y. pestis* YP043 at an MOI of 10 (*n* = 6). 20 min post-infection, macrophages were washed and the medium was replaced with medium containing 8 μg/ml gentamicin. One h later, culture medium was removed, cells were washed with 1X PBS, and fresh medium containing 2 μg/ml gentamicin was added. Bacterial numbers as a function of bioluminescence were determined every 2 h for 20 h using an IVIS Spectrum optical imager. Bioluminescent data from each time point was normalized to fold change compared to the 2 h time point by dividing each time point by the 2 h RLU reading and is displayed as mean fold change ± *S.D*. (in some cases the *S.D*. is smaller than the symbol size). Data is shown from one representative experiment of two independent experiments.

Together these data suggest that an optimized gentamicin assay for *Y. pestis* should include two doses of gentamicin. The first should be an initial dose to efficiently kill the majority of the extracellular bacteria in a short incubation period (e.g., 1 h) without inhibiting intracellular survival. The data from short term incubations with gentamicin (Figure 5) suggest that this concentration should not exceed 8 μg/ml. After the 1 h incubation, the medium should be removed as continued incubation in 8 μg/ml results in inhibition of intracellular bacteria (Figure 2). The medium should then be replaced with a lower dose that inhibits extracellular bacterial growth but not intracellular survival of the bacteria during extended incubations. The data from extended incubations with gentamicin (Figure 2) suggest that this maintenance dose should not exceed 2 μg/ml. To test these empirically derived gentamicin concentrations, peritoneal or RAW264.7 macrophages were infected with YP043, a fully virulent strain of *Y. pestis* CO-92 expressing the Lux_P*tolC*_ bioluminescent bioreporter (Sun et al., 2012). Twenty minutes later, gentamicin was added at a final concentration of 8 μg/ml. Cells were incubated for 1 h, washed with 1X PBS, and the medium was replaced with medium containing 2 μg/ml gentamicin. Intracellular bacterial survival was monitored by bacterial bioluminescence every 2 h for 20 h using an IVIS optical imager (Figure 6). In peritoneal macrophages, bioluminescence slightly decreased over the first ~8 h of infection, indicating an initial inhibition of bacterial survival by the macrophages, but then at ~12 h, bacterial bioluminescence began to increase, reaching a steady state level at 16 h that was maintained for the remainder of the infection (Figure 6A). Based on our live imaging experiments, this steady state level at later time points likely represents a balance between intracellular replication and gentamicin killing of extracellular bacteria from lysed macrophages (Figure 1). In RAW264.7 macrophages we did not observe an initial decrease in bacterial bioluminescence, supporting that this cell type is more permissive to intracellular bacterial infection (Figure 6B). Instead, we observed a ~2-fold increase in bioluminescence over the first 4 h, which plateaued at this level for the first 10 h of the infection. Then, similar to the peritoneal macrophages, at ~12 h post-infection, bacterial bioluminescence began to increase, indicating further intracellular bacterial proliferation.

## Discussion

The antibiotic protection assay has been a key technique for the study of bacterial intracellular pathogenesis. This assay was originally developed on the premise that certain classes of antibiotics are excluded from eukaryotic cells, resulting in antibiotic-mediated killing of extracellular bacteria but not intracellular bacteria. While researchers have more recently recognized that antibiotics originally thought to be excluded can inhibit the growth of several bacterial pathogens, only a few systematic studies have been performed to characterize the impact of antibiotic treatment on specific pathogens (Hand and King-Thompson, 1986; Drevets et al., 1994). We took particular notice of this in the *Yersinia* field, where large variations in antibiotic concentrations and exposure times have been reported in the literature (Pujol and Bliska, 2003; Benedek et al., 2004; Leigh et al., 2005; Ponnusamy et al., 2011; Sha et al., 2013; Spinner et al., 2014; Tiner et al., 2015; van Lier et al., 2015). We were concerned that such variations in the antibiotic protection assay between laboratories had the potential to decrease reproducibility of experiments and potentially produce artificial phenotypes due to unanticipated influence by the antibiotic. As such, our goals here were to specifically demonstrate that variations in the gentamicin protection assay could alter *Y. pestis* intracellular growth and suggest that researchers carefully consider antibiotic concentrations and exposure times when designing future intracellular experiments with *Y. pestis* (or any bacteria).

Through the use of live-cell microscopy we were able to observe *Y. pestis* interactions with macrophages in the absence of gentamicin. These observations suggest that washing of infected cells removes the majority of bacteria not phagocytosed by the macrophages, resulting in the absence of extracellular growth during the first several hours of the experiment. However, infected macrophages eventually lyse and release viable *Y. pestis* into the medium. These bacteria then replicate extracellularly. Importantly, our experience with live cell microscopy suggests that macrophage lysis is not synchronized and the timing of when macrophages lyse and initiation of extracellular growth can vary from experiment to experiment. Therefore, more conventional assays to study intracellular proliferation without microscopy will require addition of an antibiotic in the medium to ensure that extracellular growth is not mistaken for intracellular growth. However, the data reported here also suggest that researchers should carefully optimize the gentamicin protection assay to minimize potential artificial influence on the system. Using an empirical approach, we defined the maximum concentrations of gentamicin that should be used in a two-step gentamicin protection assay as 8 and 2 μg/ml (Figure 6). These concentrations represent the maximum concentrations of gentamicin that inhibit *Y. pestis* growth without demonstrating a significant impact on intracellular numbers (as compared to live microscopy in the absence of antibiotic). However, important considerations in optimizing intracellular assays are not only antibiotic concentration and exposure time, but also how *Y. pestis* is handled prior to interactions with host cells and the cell type being used. For example, while our data indicates that 1 h incubation with 8 μg/ml of gentamicin is the minimum bactericidal concentration for *Y. pestis*, this was determined for early logarithmically growing bacteria. We have observed that for stationary phase cultures, 16 μg/ml of gentamicin is required to eliminate all extracellular bacteria in 1 h (Sun et al., 2012; Connor et al., 2015). Furthermore, our data comparing peritoneal and RAW264.7 macrophages support that researchers should not assume that empirically determined gentamicin concentrations that do not influence intracellular growth in one cell type will not alter growth in another. Wendte et al. also recognized differences in intracellular *Y. pestis* sensitivity to gentamicin between THP-1 and RAW264.7 macrophages (Wendte et al., 2011). Therefore, different macrophages may require concentrations different from those used here. In summary, we hope these studies help researchers reconsider the potential impact of gentamicin on *Y. pestis* intracellular growth when designing future experiments using the antibiotic protection assay.

## Ethics statement

This study was carried out in accordance with the recommendations of the University of Louisville Institutional Animal Care and Use Committee (IACUC). The protocol was approved by the IACUC.

## Author contributions

Conceptualized experiments: TV, ML. Performed experiments: TV, AP, MC, JW. Analyzed data: TV, JW, ML. Drafted and reviewed manuscript: TV, AP, MC, ML.

### Conflict of interest statement

The authors declare that the research was conducted in the absence of any commercial or financial relationships that could be construed as a potential conflict of interest.

## Supplementary material

Supplemental Figure 1 Minimum inhibitory concentration of gentamicin for KIM D19 *Y. pestis*. Logarithmically growing KIM D19 *Y. pestis* pCD1^(−)^ Lux_P*tolC*_ was diluted to 1.5 × 10^6^ CFU per well in a 96 well plate containing gentamicin at the indicated concentrations. One h after inoculation into gentamicin, bacteria were removed, serially diluted in 1x PBS, and enumerated on agar to determine bacterial viability after exposure to gentamicin (*n* = 3). Each point represents one sample and the bars represent the mean CFU. The dotted lines indicate the limit of detection. Data is shown from one representative experiment of three independent experiments. ANOVA with Dunnett's *post-hoc* analysis compared to 0 μg/ml gentamicin: ^**^ *p* ≤ 0.01; ^****^*p* ≤ 0.0001; ns = not significant.

Supplemental Movie 1 5 × 10^5^ peritoneal macrophages were infected with *Y. pestis* YPA165 expressing mCherry [pCD1^(+)^] at a MOI = 3. One h after infection, cells were washed to remove extracellular bacteria and macrophages were imaged by live confocal microscopy. Images were taken every 20 min beginning 1 h post-infection and continuing for 24 h. One representative field from four fields imaged is shown.

Supplemental Movie 2 5 × 10^5^ peritoneal macrophages infected with *Y. pestis* YPA081 expressing EGFP [pCD1^(−)^] at a MOI = 3. One h after infection, cells were washed to remove extracellular bacteria and macrophages were imaged by live confocal microscopy. Images were taken every 20 min beginning 1 h post-infection and continuing for 24 h. One representative field from four fields imaged is shown.
