# Supplementary figures and images for: Impact of Gentamicin Concentration and Exposure Time on Intracellular Yersinia pestis
Source: Front Cell Infect Microbiol. 2017 Dec 11;7:505. doi: 10.3389/fcimb.2017.00505 (PMC5732358; doi:10.3389/fcimb.2017.00505)

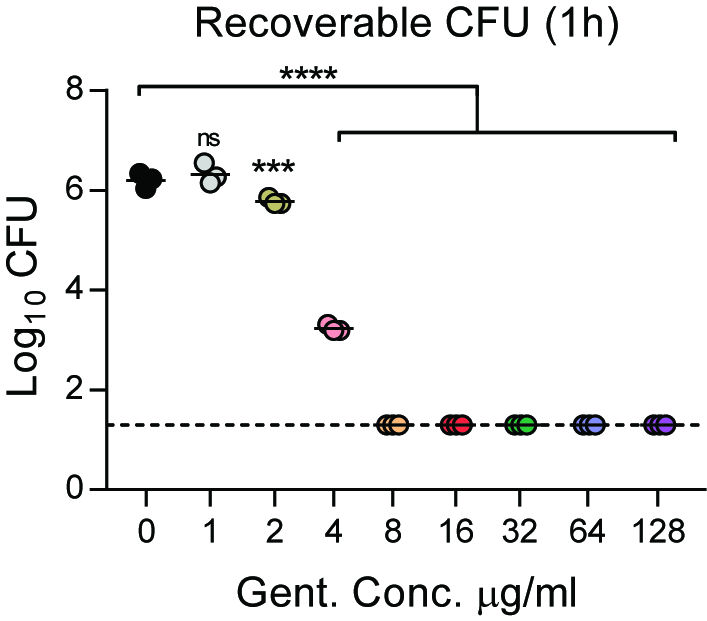

Supplement: Supplemental Figure 1 — Minimum inhibitory concentration of gentamicin for KIM D19 Y. pestis. Logarithmically growing KIM D19 Y. pestis pCD1(−) LuxPtolC was diluted to 1.5 × 10^6^ CFU per well in a 96 well plate containing gentamicin at the indicated concentrations. One h after inoculation into gentamicin, bacteria were removed, serially diluted in 1x PBS, and enumerated on agar to determine bacterial viability after exposure to gentamicin (n = 3). Each point represents one sample and the bars represent the mean CFU. The dotted lines indicate the limit of detection. Data is shown from one representative experiment of three independent experiments. ANOVA with Dunnett's post-hoc analysis compared to 0 μg/ml gentamicin: ** p ≤ 0.01; ****p ≤ 0.0001; ns = not significant. [file Image1.TIF]
